# Clinical impact of measurable residual disease monitoring by ultradeep next generation sequencing in *NPM1* mutated acute myeloid leukemia

**DOI:** 10.18632/oncotarget.26400

**Published:** 2018-11-27

**Authors:** Nikhil Patkar, Rohan Kodgule, Chinmayee Kakirde, Goutham Raval, Prasanna Bhanshe, Swapnali Joshi, Shruti Chaudhary, Y. Badrinath, Sitaram Ghoghale, Shraddha Kadechkar, Syed Hasan Khizer, Sadhana Kannan, Dhanalaxmi Shetty, Anant Gokarn, Sachin Punatkar, Hasmukh Jain, Bhausaheb Bagal, Hari Menon, Manju Sengar, Navin Khattry, Prashant Tembhare, Papagudi Subramanian, Sumeet Gujral

**Affiliations:** ^1^ Haematopathology Laboratory, ACTREC, Tata Memorial Centre, Navi Mumbai, India; ^2^ Adult Haematolymphoid Disease Management Group, Tata Memorial Centre, Mumbai, India; ^3^ Biostatistics, ACTREC, Tata Memorial Centre, Navi Mumbai, India; ^4^ Dept of Cytogenetics, ACTREC, Tata Memorial Centre, Navi Mumbai, India; ^5^ Homi Bhabha National Institute, Training School Complex, Mumbai, India; ^6^ Haemato-Oncology, CyteCare Cancer Hospital, Bangalore, India

**Keywords:** acute myeloid leukemia, NPM1, measurable residual disease, next-generation sequencing, multiparameter flow cytometry

## Abstract

Detection of measurable residual disease (MRD) by mutation specific techniques has prognostic relevance in *NPM1* mutated AML (*NPM1*^mut^ AML). However, the clinical utility of next generation sequencing (NGS) to detect MRD in AML remains unproven. We analysed the clinical significance of monitoring MRD using ultradeep NGS (NGS-MRD) and flow cytometry (FCM-MRD) in 137 samples obtained from 83 patients of *NPM1*^mut^ AML at the end of induction (PI) and consolidation (PC). We could monitor 12 different types of *NPM1* mutations at a sensitivity of 0.001% using NGS-MRD. We demonstrated a significant correlation between NGS-MRD and real time quantitative PCR (RQ-PCR). Based upon a one log reduction between PI and PC time points we could classify patients as NGS-MRD positive (<1log reduction) or negative (>1log reduction). NGS-MRD, FCM-MRD as well as *DNMT3A* mutations were predictive of inferior overall survival (OS) and relapse free survival (RFS). On a multivariate analysis NGS-MRD emerged as an independent, most important prognostic factor predictive of inferior OS (hazard ratio, 3.64; 95% confidence interval [CI] 1.58 to 8.37) and RFS (hazard ratio, 4.8; 95% CI:2.24 to 10.28). We establish that DNA based *NPM1* NGS MRD is a highly useful test for prediction of relapse and survival in *NPM1*^mut^ AML.

## INTRODUCTION

Recent developments in sequencing cancer genomes have advanced our understanding of the complex molecular landscape of AML. Even after addressing the extensive network of cytogenetic abnormalities, molecular heterogeneity, epigenetic and gene expression profiles in AML, the prognostic stratification in AML mainly relies upon only cytogenetics and a handful of gene mutations. Subsequent to risk stratification and standard treatment, the 5 year survival is less than 60% in adult AML [1]. Therefore, there is a clinical need to develop assays that identify patients who have a suboptimal response to therapy and are at a high risk of relapse so that they can be treated with intensive post remission strategies such as allogeneic bone marrow transplantation. The detection of leukemic cells at a threshold below the morphologic limit of detection is called measurable residual disease (MRD). Studies over the last two decades have shown that the presence of MRD is an important prognostic factor that is highly predictive of outcome in a variety of haematological malignancies. Today, MRD detection is routinely performed for assessment of response to therapy as well as guiding post remission strategies in chronic myeloid leukemia and precursor B lineage acute lymphoblastic leukemia. MRD can be measured by sensitive real-time PCR for AMLs that harbour gene fusions (for e.g. *RUNX1-RUNX1T1*). However, for the majority of AML, immunophenotyping (FCM-MRD) remains as the most accepted method to detect MRD [2–4].

Acute myeloid leukemia (AML) with mutated *NPM1* is a specific subtype of AML with a recurrent genetic abnormality and favourable outcome [5]. *NPM1*^mut^ AML is one of the most common subsets of AML in adults and comprises around 35% overall adult AML cases and around 45% of normal karyotype AML [6, 7]. More than 50 types of insertion mutations have been detected in exon 12 of the *NPM1* gene [8]. These mutations are specific to the blast compartment and are not present in mature myeloid and lymphoid cells, deeming *NPM1* as reliable marker for MRD [9]. For MRD monitoring of *NPM1*^mut^ AML, the use of molecular techniques has been more prevalent. Recent studies have shown that serial monitoring of *NPM1* mutations from the blood or bone marrow during chemotherapy is highly predictive of relapse. Majority of these studies have been RNA based and have used mutation specific approaches for the detection of MRD [10–12]. A disadvantage of a mutation specific approach is that the *NPM1* mutation must be characterized at diagnosis by sequencing and each type of *NPM1* mutation must be validated as an independent MRD assay. This is further complicated by varying performance characteristics of mutation specific primers and fluorescent probe combinations.

DNA based next generation sequencing (NGS) is a scalable solution that has the potential for detection of MRD in AML [13–15]. As an NGS based assay can cover all occurring mutations in a specific genomic locus, it can potentially overcome the technical drawbacks of real time quantitative PCR (RQ-PCR) and provide a unique solution for detection of MRD. However, studies that have evaluated the clinical impact of NGS-MRD are largely lacking. In this study, we have validated an ultra-sensitive technique for *NPM1*^mut^ AML that can detect *NPM1* mutations at a frequency of 0.001%. We have then compared NGS-MRD with FCM-MRD, the gold standard for detection of MRD in AML as well as RQ-PCR for the commonly occurring type A *NPM1* mutations. We demonstrate that NGS-MRD for *NPM1*^mut^ AML is an independent prognostic factor significantly predictive of outcome in *NPM1*^mut^ AML.

## RESULTS

### Patient outcome

The mean overall survival (OS) was 40.02 months (95%CI 33.73 to 46.32) for the entire cohort (median not reached). The mean relapse free survival (RFS) was 33.31 months (95%CI 26.85 to 39.77), median RFS was 19.43 months (95%CI 14.27 to 43.03) months (Supplementary Figure 1). The median follow-up was 23.5 months. Additional patient characteristics can be seen in Supplementary Table 1A and 1B.

### Types of NPM1 mutations monitored & clinical relevance of NGS-MRD

**Figure 1:**
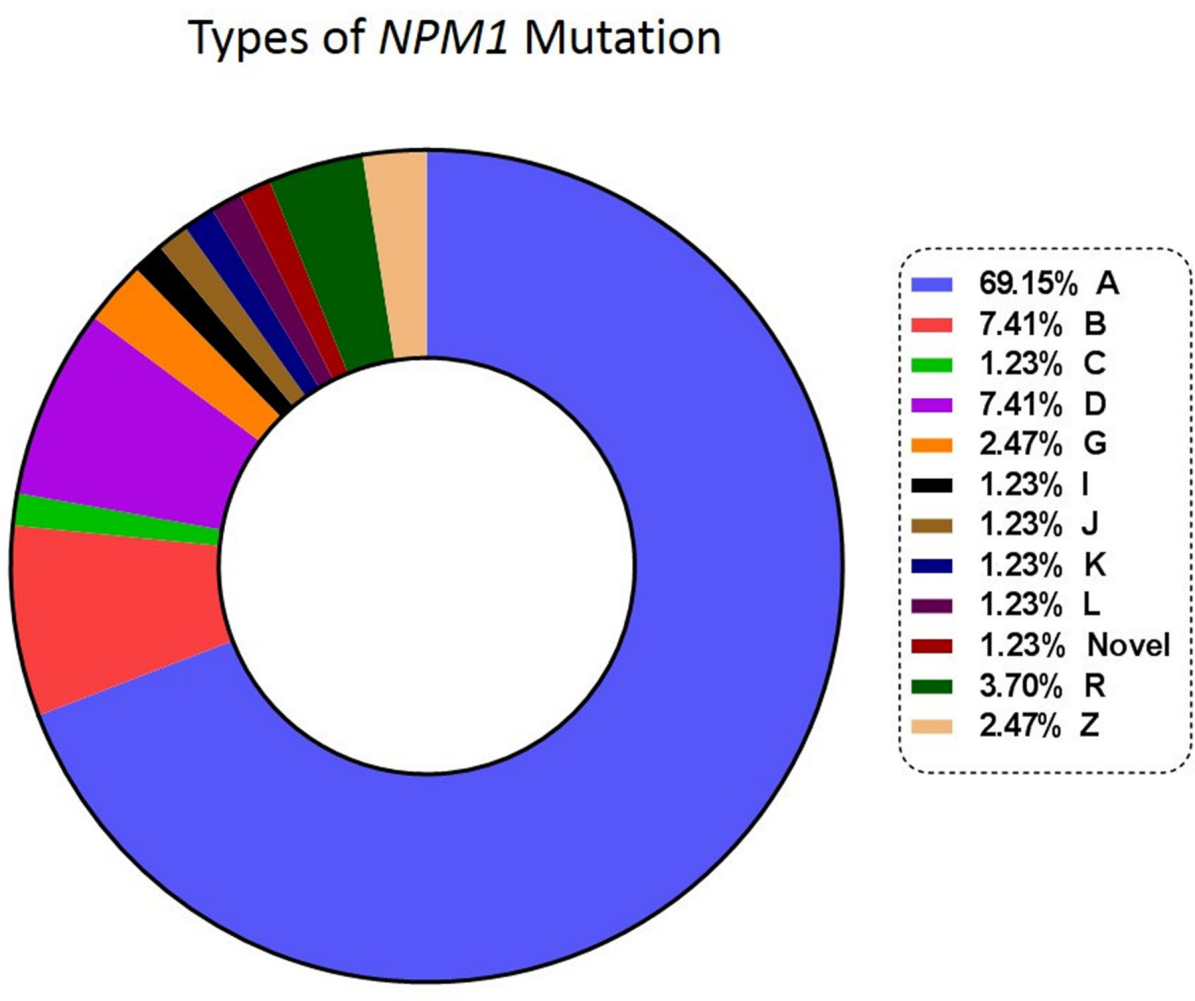
Types and frequencies of *NPM1* mutations

**Figure 2:**
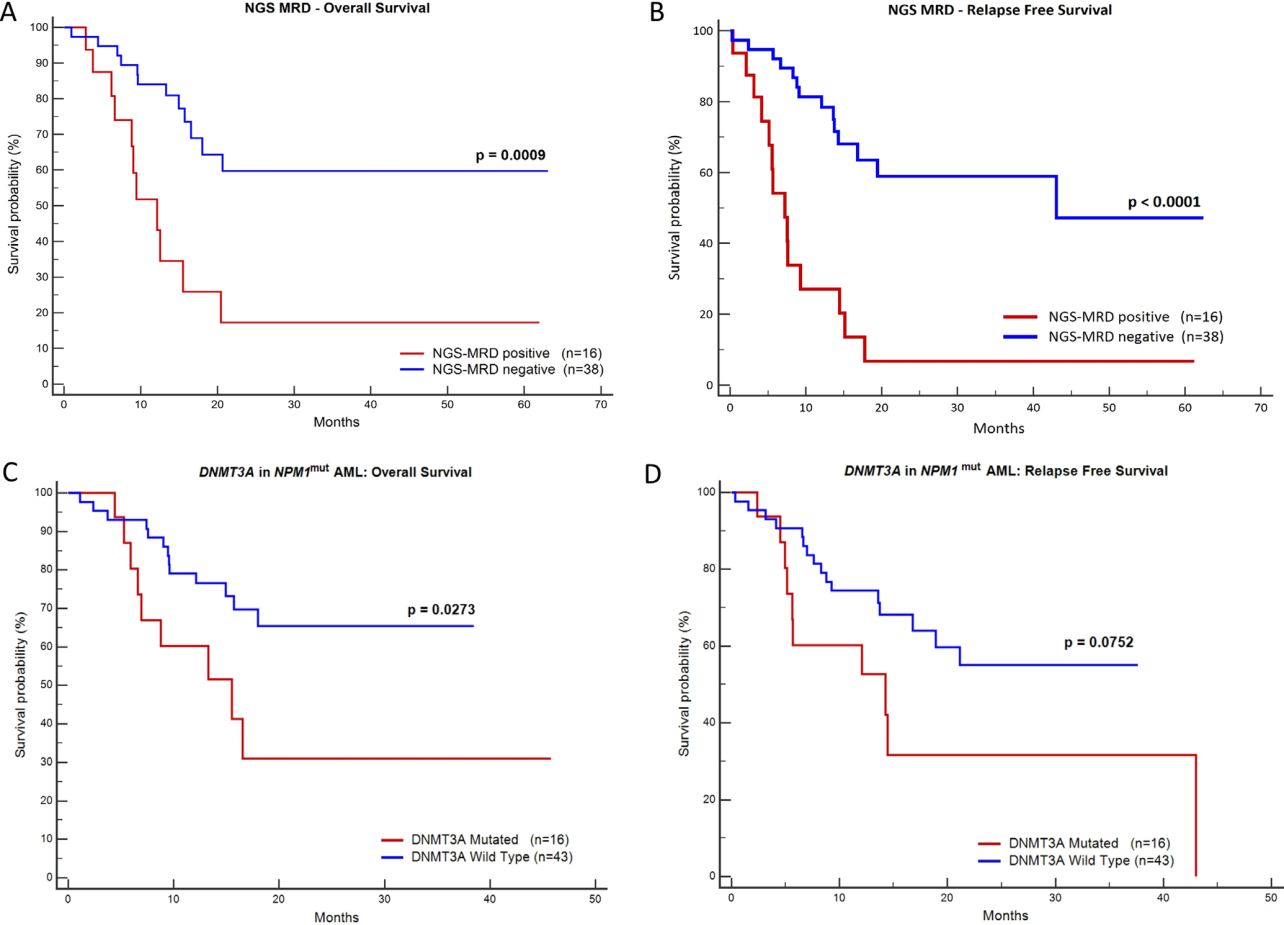
Kaplan-Meier graphs of NGS-MRD and presence of *DNMT3A* mutation Plots (**A**) and (**B**) demonstrate that the presence of NGS MRD is highly predictive of inferior OS and RFS. The plots (**C**) & (**D**) show that presence of *DNMT3A* mutation is predictive of the inferior OS (C) and likely RFS (D).

Diagnostic DNA was available in 81 patients. These samples were sequenced to characterize the mutation, ensure the stability of *NPM1* mutation over post-induction (PI) and first post consolidation (PC) time points and ascertain whether there was any switch in the mutation type in the course of treatment [16]. We did not detect a clonal switch in our cohort. Figure 1 shows the types of *NPM1* mutations and their frequencies. Type A, B and D were the most common subtypes (83.95%, Supplementary Table 2). On comparing the prognosis of these common subtypes against the other uncommon subtypes no significant difference in OS and RFS could be observed. We also detected a novel 4 bp insertion, c.873_874insGCCA. Based on a one log reduction between PI and PC time points, out of 54 cases tested, 16 (29.63%) were NGS-MRD positive and 38 (70.37%) were NGS-MRD negative. Presence of NGS-MRD was significantly predictive of an inferior OS and RFS (Figure 2 and Table 1).

**Table 1:** Prognostic significance of MRD in *NPM1*^mut^ AML by univariate and multivariate Cox analysis

Univariate Cox analyses				
	Overall Survival (OS)		Relapse Free Survival (RFS)	
	HR (95% CI)	*P*	HR (95% CI)	*P*
**NGS MRD**		***0.002***		***0.0001***
MRD Negative	1		1	
MRD Positive	3.74 (1.64 to 8.54)		4.5 (2.2 to 9.86)	
**PI FCM-MRD**		***0.0081***		***0.012***
MRD Negative	1		1	
MRD Positive	2.6 (1.29 to 5.27)		2.3 (1.2 to 4.26)	
**PC FCM-MRD**		0.26		0.5
MRD Negative	1		1	
MRD Positive	1.84 (0.65 to 5.30)		1.42 (0.51 to 3.92)	
***FLT3*-ITD**		0.83		0.96
*FLT3*-ITD Negative	1		1	
*FLT3*-ITD Positive	1.08 (0.53 to 2.23)		1.02 (0.53 to 1.94)	
***DNMT3A***		**0.0330**		0.08
*DNMT3A* Negative	1		1	
*DNMT3A* Positive	2.53 (1.08 to 5.93)		2.08 (0.92 to 4.70)	

Multivariate Cox analyses				
	Overall Survival (OS)		Relapse Free Survival (RFS)	
	HR (95% CI)	*P*	HR (95% CI)	*P*
**NGS MRD**	3.64 (1.58 to 8.37)	***0.0025***	4.8 (2.24 to 10.28)	***0.0001***
**PI FCM-MRD**	2.61 (1.14 to 6.00)	***0.0246***	2.71 (1.27 to 5.8)	***0.0105***

Abbreviations: HR, hazards ratio; CI, confidence interval; ITD, internal tandem duplication.

### Comparison of MRD results obtained by NGS (VAF) and RQ-PCR (only for Type A mutant NPM1)

To compare NGS-MRD results using an orthogonal technique, we used a DNA based RQ-PCR based assay to detect MRD for Type-A *NPM1* mutations at end of PI and PC. RQ-PCR was done in total 71 samples carrying Type-A *NPM1* mutation at PI (44) and PC (27) time points. Values obtained from RQ-PCR were not significantly different from corresponding NGS variant allelic frequency (VAF) (*p* = 0.7134; Supplementary Figure 2). A significant concordance was seen between NGS-VAF and RQ-PCR data for MRD samples with minimal bias (*r*^2^ = 0.9375, *p* < 0.0001; Supplementary Figure 2). NGS-MRD was more sensitive as compared to RQ-PCR. Ten samples were positive by NGS-MRD (range 0.0016 to 0.018, median = 0.0045) and RQ-PCR negative. Similarly, only 3 samples were RQ-PCR positive but NGS-MRD negative.

### Relevance of DNMT3A mutations in NPM1^mut^ AML

A total of 59 *NPM1*^mut^ AML samples with optimum DNA concentration at baseline were subjected for analysis of *DNMT3A* mutation. For baseline samples median coverage was 987x. Out of 59 patients tested, 16 (26.6%) were positive for *DNMT3A* mutation with VAF ranging from 3.5% to 50%. The arginine 882 hotspot was the most commonly affected amino acid in our cohort (43.75%). Presence of *DNMT3A* mutations in *NPM1*^mut^ AML were predictive of inferior OS (*p* = 0.027) and possibly inferior RFS (*p* = 0.075; Figure 2). This agrees with other studies where *DNMT3A* mutations have been reported to have adverse prognosis in *NPM1*^mut^AML [10, 17, 18].

### Dynamics of VAF changes in DNMT3A and comparison with NPM1-VAF

**Figure 3:**
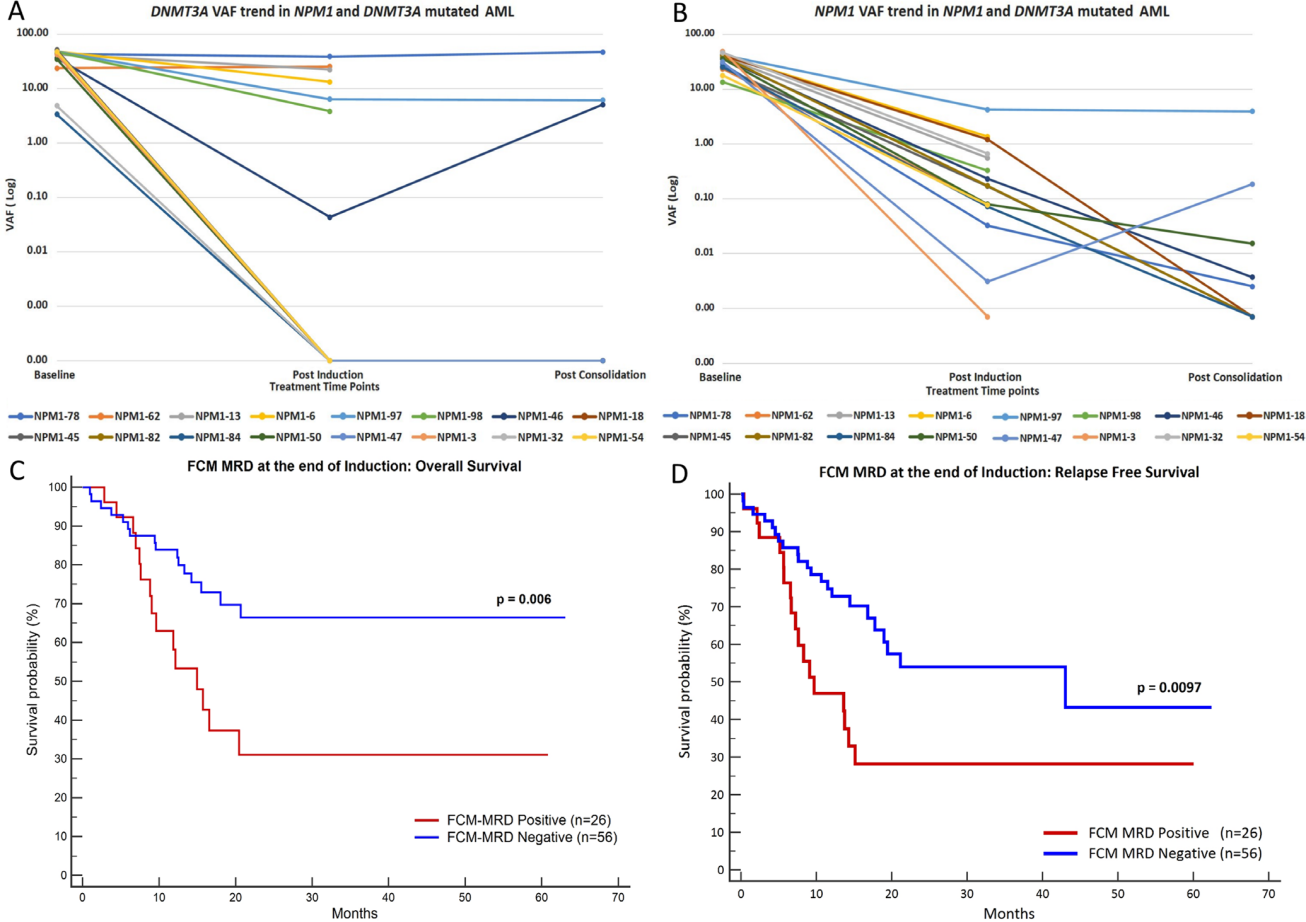
Plot (**A**) and (**B**) represent the trends of VAF during treatment for *DNMT3A* and *NPM1* in dual mutated patients. Plots (**C**) and (**D**) show Kaplan-Meier graphs of FCM-MRD, depicting that FCM-MRD at the end of induction is predictive of inferior OS (C) and RFS (D).

The details of *DNMT3A* mutations and the dynamics of these mutations at post therapeutic time points can be seen in Supplementary Table 3. In 7 out of 16 cases, *DNMT3A* mutations were persistent at the end of induction and 3 cases (out of 7 tested) at end of consolidation. The corresponding *NPM1* VAF can be also seen in Supplementary Table 3 and Figure 3.

### Clinical relevance of FCM-MRD

At the end of induction, FCM-MRD was detected in 26 out of 82 (31.7%) patients at levels ranging from 0.02 to 6.32% (median: 0.7%). Similarly, at end of consolidation FCM-MRD was detected in 8 out of 40 (20%) patients at levels ranging from 0.01 to 1.8% (median: 0.05%). The presence of FCM-MRD at the end of induction was highly predictive of inferior OS as well as and RFS (Figure 3). However, FCM-MRD measured at the PC time point did not influence either OS or RFS as can be seen by results of univariate analysis in Table 1.

### Comparison of MRD results obtained by NGS (VAF) and FCM-MRD values

A total 82 cases at the PI timepoint and 40 at PC timepoint were analyzed for MRD using FCM and NGS. A comparison of these results can be seen in Supplementary Figure 3. MRD values measured using these two modalities correlated poorly (*r* = 0.1685, *p* = 0.07).

### Multivariate analysis

On a multivariate Cox proportional hazards regression analysis, the presence of NGS-MRD was an independent factor predictive of inferior OS and RFS. In fact, NGS-MRD and PI FCM-MRD were the only significant prognostic factors for relapse and death (Table 1). NGS-MRD had a higher hazard ratio as compared to FCM-MRD.

## DISCUSSION

*NPM1* gene mutations are the largest subset of molecular alterations seen in adult AML. It is therefore imperative that we develop effective strategies for monitoring MRD. Most of the molecular techniques that have been published for MRD detection in *NPM1*^mut^ MRD are based on quantitative measurement of *NPM1*-mutant transcripts [10, 17–20] or DNA based RQ-PCR for the detection of mutant *NPM1* alleles [2, 17, 21]. Recently, droplet digital PCR has also been used as tool to measure MRD in *NPM1*^mut^ AML [12, 22]. The importance of sequential monitoring of *NPM1* mutations has been described by a few cohorts [10, 20, 21]. These papers have established the utility of RNA based MRD detection in *NPM1*^mut^ AML. In fact, Ivey and colleagues indicated that real time PCR based *NPM1* MRD status was the only important predictor of relapse or death [10]. They conclusively showed that *NPM1* mutations are a reliable marker for monitoring disease progression. There are very few studies that have evaluated the utility of molecular *NPM1*-MRD in comparison with immunophenotypic MRD. Here, we demonstrate that at the early time-points of therapy (PI) FCM-MRD is important in prediction of relapse. As seen in Supplementary Table 4, FCM-MRD positive patients had a shorter OS and RFS as compared to FCM-MRD negative patients.

DNA based NGS assays overcome many of the obstacles of real time PCR as they do not need patient specific sequence information prior to monitoring. In our dataset, even though majority of the mutations were of type A, B and D, we detected nine uncommon *NPM1* mutation subtypes (Figure 1, Supplementary Table 2). These patients could have been missed by RQ-PCR assays that target common types of *NPM1* mutations (type A, B and D). Our dataset shows the importance of a generalized approach to the detection of MRD in *NPM1*^mut^ AML. The fact that we could identify and monitor rare *NPM1* mutations using this technique validates our approach. There have been reports of switch of *NPM1* mutations at relapse [16]. Although, we did not find any evidence of switch in the *NPM1* mutation type either at post induction or post consolidation time points, an NGS based approach provides a definitive advantage over mutation specific testing in potentially detecting a *NPM1* clonal switch. There have been contradictory observations in the literature regarding the prognosis of uncommon *NPM1* mutation as against the common subtypes [23, 24]. In our cohort, we did not find significant difference between common and uncommon subtypes.

Recent data has shown that NGS based MRD assays are potentially more sensitive than flow cytometry and offer comparable sensitivity as RQ-PCR [13, 25–28]. Based on a 1-log change in VAF between PI and PC time points, patients were categorised into NGS-MRD negative and NGS-MRD positive. Failure to achieve a 1-log reduction at PC time point predicted shorter relapse free and overall survival. Thus, this criterion can be used prospectively in determination of MRD in *NPM1*^mut^ AML patients. As seen in Supplementary Table 4, NGS-MRD positive patients had worse OS and RFS as compared to FCM-MRD positive patients.

In our cohort, significant number of patients who were FCM-MRD-negative had detectable MRD levels using deep sequencing. This resulted in a poor correlation between FCM-MRD and NGS-MRD VAF (Supplementary Figure 3). Unlike precursor B lineage acute lymphoblastic leukaemia which has a high frequency of leukemia associated immunophenotype (LAIP), AML MRD detection by flow cytometry is inherently complex. This is because FCM-MRD detection in AML is conceptually different from B or T-ALL because of lack of a common identifier for abnormal myeloid blasts [29, 30]. An inherent problem with FCM-MRD for AML is inability to identify leukemic populations below the threshold of 0.1% with a high level of confidence [31]. Furthermore, FCM-MRD detection in AML is difficult when the leukemia associated immunophenotype (LAIP) is expressed only by the subpopulation of leukemic cells, as most of the leukemic cells have similar immunophenotype compared to normal population. LAIP may change during the treatment, thereby making initial MRD markers irrelevant in subsequent time points [32–34]. It has been proven that deep sequencing based MRD has a higher sensitivity as compared to FCM-MRD in *NPM1*^mut^ AML [13, 35]. In our study NGS MRD had a one log higher sensitivity in dilution experiments. Recently, Malmberg et al. demonstrated a consistent lower estimation of leukemic cell burden using FCM-MRD compared to targeted deep sequencing. MRD was even detected using targeted deep sequencing in cases where FCM-MRD was below the level of LOD [36]. These reasons may be an explanation for poor correlation between FCM and NGS based MRD observed in our study.

We saw agreement with previous studies that *NPM1* mutations often co-occur with *DNMT3A* mutations and portend a poor prognosis [37–39]. Furthermore, we detected persistence of these *DNMT3A* mutations in 7 post treatment samples even when there was significant reduction in the *NPM1* mutant levels. These are likely to be associated with age related clonal haematopoiesis [40, 41]. It has been reported that the presence of *FLT3*-ITD and high allelic ratio of *FLT3*-ITD is a poor prognostic factor in *NPM1*^mut^ AML [4, 42]. However we could not confirm these findings in our patients.

We found a reasonable correlation between paired bone marrow and peripheral blood *NPM1* mutant VAF in 19 samples (see Supplementary Methods for equivalence studies, Supplementary Figure 4). It has previously been demonstrated that monitoring of *NPM1* mutant levels can be done using peripheral blood or plasma [10, 20, 43, 44]. A blood sample can be obtained easily as opposed to a painful bone marrow aspiration, however, there is a risk of missing very low level mutations in peripheral blood samples [45]. Such a recommendation has also been made by ELN MRD working party [46]. Nonetheless, to negate a bias from inclusion of blood samples, we evaluated the prognostic relevance of sequential monitoring of *NPM1* NGS-MRD in only bone marrow samples (Supplementary Methods, Supplementary Figure 5). Thus, we can conclude that, patients can be sequentially monitored using bone marrow as well as blood during post treatment time points for *NPM1* NGS MRD.

Additional somatic mutations that are known to occur in *NPM1* mutated AML could not be monitored using this assay [10, 24]. As this assay focusses only on the *NPM1* mutation for calculating AML MRD, even though rare; clonal evolution leading to relapse because of the non-*NPM1* mutations could not be detected [44, 47]. Another potential limitation of the original assay [25] was the possibility of barcode cross contamination leading to a misattribution of the samples contaminating the library and thereby obtaining inaccurate results. To avoid this problem we incorporated a dual indexing strategy [48]. The latter is used routinely in sample multiplexing and allows detection of rare mutations in multiplexed samples with minimal errors.

To summarize, this is one of the few papers that has evaluated the clinical relevance of NGS MRD in *NPM1*^mut^ AML. Shayegi et al. used the IonTorrent PGM chemistry to deep sequence *NPM1* gene for mutations in a small subgroup of 10 patients using genomic DNA as a template [49]. They concluded that an RNA expression value of 1% *NPM1* mutant/*ABL* corresponds to a value of 0.016% *NPM1* mutant alleles on genomic DNA (per *NPM1* wild-type alleles). However, we could not confirm the clinical relevance of using such a cut-off in our dataset at either PI or PC time points. The results of Shayegi and colleagues as well as our results need to be confirmed by larger cohorts for incorporation into treatment algorithms.

## MATERIALS AND METHODS

### Patients

A total of 83 *NPM1*^mut^ AML were accrued into the study after informed consent from October 2012 to May 2017 (after exclusion of patients with induction deaths and refractory disease). Patients were diagnosed as per current WHO 2016 criteria. *NPM1* mutations were detected initially using fragment length analysis [50] and subsequently characterized using the NGS assay (see below). These patients were treated with standard 3 + 7 induction followed by 3 cycles of high dose cytarabine (12–18g/m^2^) and followed up till January 2018. FCM-MRD was assessed from the bone marrow at the end of induction (PI) (BM, *n* = 82) and at the end of first consolidation (PC, *n* = 40). NGS-MRD was assessed from 82 PI samples (all BM) and 55 PC samples, of which 13 samples were sourced from the peripheral blood (PB). In total, there were 54 paired samples between PI and PC time points. (Supplementary Figure 6). *FLT3*-ITD was detected on the diagnostic sample by fragment length analysis [50].

### Detection of MRD using NGS in NPM1^mut^ AML

We adopted a strategy as described by Salipante et al. with minor modifications as detailed below [25]. Briefly the assay incorporated a one-step strategy wherein, Illumina adapter linked locus specific primers were designed for amplification of exon 12 of *NPM1* (Integrated DNA Technologies (Coralville, IA, USA)). To prevent contamination from other sample indices, we used a dual indexing strategy with 10 bp sample specific index barcode in both forward and reverse primers. The primer sequences are as listed in Supplementary Table 5.

### Assay setup

Assay setup was as follows: For a total reaction volume of 75 μl, 37.5 μl of NEBNext High-Fidelity 2X PCR Master Mix (New England BioLabs Inc., Massachusetts, USA), and 10 uM each forward and reverse primers were used to amplify 600ng of genomic DNA (approximately 1,00,000 genomic cell equivalents). PCR cycling conditions were as follows: Initial denaturation of 95°C for 15 minutes; then 35 cycles of denaturation at 94°C for 1 minute, annealing at 65°C for 1 minute, and extension at 72°C for 1 minute; followed by extension cycle of 72°C for 45 seconds. PCR products were size selected using Agencourt AMPure XP beads (Beckman Coulter Inc., California, USA) and quantified using Qubit dsDNA HS assay (Thermo Fisher Scientific, Massachusetts, USA). Samples were deep sequenced after pooling in equimolar concentration on an Illumina MiSeq (Illumina, San Diego, CA, USA) next generation sequencer using a 150 bp paired end V2 chemistry.

### Data analysis

Runs were demultiplexed using the MiSeq onboard software. Paired-end reads were self-assembled using PANDAseq [51]. Self-assembled reads were mapped to the human genome (GrCh37) using bwa v0.7.12 [52]. Samtools v0.1.19 [53] was used to sort the bam file and create a mpileup file. Variant indels were called using VarScan v2.3.7 [54]. Finally, once the .vcf file was generated, it was annotated using an internal database and custom scripts. For additional redundancy, we also introduced a synthetic control in each sequencing dataset. This ‘bioinformatics control’ with 20 bp insertion served as mechanism to ensure that the pipeline worked as expected. To make a variant call, a minimum of two mutant reads had to be obtained.

### NPM1 NGS-MRD assay validation

We increased the analytical sensitivity of the original NGS-MRD assay (as described by Salipante) to detect *NPM1* mutations at a high sensitivity of 0.001%. A limit of detection experiment demonstrated that the assay could detect the *NPM1* mutation at 1:100,000 frequency. To ensure that the assay did not make false *NPM1* insertion calls we performed a limit of blank experiment on 30 normal DNA samples. The details pertaining to these experiments can be seen in Supplementary Methods.

### Controls

To account for systemic drift, we assayed two (high and low level) precision controls (OCI-AML3 cell line diluted in normal DNA at 0.2 and 0.02% VAF respectively) in every run. The results of these experiments can be seen in Supplementary Methods. In addition, we also used a NA12878 control DNA as a negative MRD control.

### Calculation and reporting of NGS-MRD

*NPM1* variant allele frequencies obtained after ultradeep sequencing at PI and PC time points were used to calculate log reduction values. (Supplementary Methods) ROC curves of log reduction values were used to calculate the cut off value that provided the optimal classification of patients in terms of overall survival with the help of Youden’s index. We determined that a 1-log reduction represented the optimal sensitivity and specificity with the corresponding Youden’s index of 0.42. We used a 1-log reduction between PI and PC time points to classify patients as NGS-MRD positive (<1-log reduction) or negative (>1-log reduction). Cases in which PI and PC values were both zero were noted as NGS-MRD negative.

### Assessment of DNMT3A mutations in NPM1^mut^ AML

We designed 61 single molecule molecular inversion probes (smMIPS) to construct a library that spanned the entire *DNMT3A* coding region. These libraries were balanced to ensure similar capture efficiencies of targeted regions as described previously [55]. Sequencing was done on an Illumina MiSeq (Illumina, San Diego, CA, USA) using the V2-300 cycle chemistry. Data was analysed using a custom pipeline that incorporates adapter trimming using ea-utils [56], read self-assembly using PEAR [57], alignment to the human genome (build hg19) using bwa (v0.7.12) [52], pre-processing of aligned files using samtools (v. 0.1.19) [53] & GATK v3.8 [58]. Variant calling was done using Varscan (v. 2.3) [54], Mutect 2.0 [58], and Platypus v0.81 [59]. Using annovar [60] the variants were annotated with population frequency databases as well as the COSMIC (v.84) [61] database.

### DNA based RQ-PCR assay for Type-A NPM1^mut^ AML

DNA extracted from OCI-AML3 cell lines carrying Type-A *NPM1* mutation were subjected to PCR to amplify exon 12 of *NPM1*. PCR products were purified and cloned into a pJET1.2/blunt cloning vector (ThermoFisher Scientific, Foster City, CA, USA) following the manufacturer’s instructions. Plasmids carrying mutant and wild type *NPM1* were confirmed by sanger sequencing, linearized and used to generate standard curves in further RQ-PCR experiments. MRD detection for type-A *NPM1* mutations using DNA as a template was done using a previously published protocol on a LightCycler 96 (Roche, Basel, Switzerland) [21].

### Detection of MRD using FCM (FCM-MRD) in NPM1^mut^ AML

Patients accrued from March 2012 to February 2015 (25 cases) had been acquired on an 8 colour BD FACSCanto II or two 10 colour BC Navios instruments using a three tube 8 colour MRD assay as seen in Supplementary Table 6. Subsequently diagnostic and follow up samples of 57 patients were acquired on two 10 colour BC Navios instruments using a two tube 10 colour MRD assay as seen in Supplementary Table 7. An identical panel was used for diagnostic sample, post induction and post consolidation time points. More than 500,000 events were acquired per tube with the 3-tube assay and 1.6 million events per tube obtained per tube with the 2 tubes, 10 colour assays. Kaluza software (v1.2) was used to analyse the. fcs/. lmd files. Boolean gating was used to focus on progenitors, monocytes while excluding lymphocytes and hematogones [62]. The focus of analysis was to study progenitor cells as they matured into monocytes and myeloid cells. MRD was calculated as a percentage of abnormal leukemic cells per total nucleated cells. Normal templates were periodically updated (once in 2 weeks). Additional experiments pertaining to the FCM-MRD assay (limit of dilution and example of approach to FCM-MRD detection) can be seen in Supplementary Methods.

### Survival analysis

Overall survival (OS) was defined as time from start of induction therapy to time of last follow up or death. Relapse free survival (RFS) was calculated from date of achievement of remission to the date of relapse, if not, death from any cause. Patient who did not relapse and were alive were censored on the last follow up date [63]. Results of the FCM-MRD and NGS-MRD assays were analysed for their impact on OS and DFS using the Kaplan-Meier technique and compared using log-rank test. Cox proportional-hazards regression was used to calculate hazard ratios assessing the prognosis of different variables with univariate and multivariate analysis. MedCalc Statistical Software version 14.8.1 (MedCalc Software, Ostend, Belgium) and SPSS (IBM Corp. Released 2015. IBM SPSS Statistics for Windows, Version 23.0. Armonk, NY: IBM Corp.) were used to perform the statistical analysis.

## CONCLUSIONS

We have evaluated the clinical utility of an ultrasensitive NGS assay for sequential monitoring of *NPM1* mutation. This assay has a prognostic impact and can be used in the prospective monitoring of *NPM*1^mut^ AMLs. It has advantages as compared to FCM-MRD in terms of a lower cost of testing, stability of DNA for shipped samples, higher sensitivity and easier interpretation. In addition, it is comparable to established RQ-PCR assay based MRD results with definite advantages. We feel that in view of the high sensitivity of the NGS assay, monitoring of *NPM1* MRD from the peripheral blood is feasible.

## SUPPLEMENTARY MATERIALS FIGURES AND TABLES


