# Correction: Clinical impact of measurable residual disease monitoring by ultradeep next generation sequencing in *NPM1* mutated acute myeloid leukemia

**DOI:** 10.18632/oncotarget.26610

**Published:** 2019-01-15

**Authors:** Nikhil Patkar, Rohan Kodgule, Chinmayee Kakirde, Goutham Raval, Prasanna Bhanshe, Swapnali Joshi, Shruti Chaudhary, Y. Badrinath, Sitaram Ghoghale, Shraddha Kadechkar, Syed Hasan Khizer, Sadhana Kannan, Dhanalaxmi Shetty, Anant Gokarn, Sachin Punatkar, Hasmukh Jain, Bhausaheb Bagal, Hari Menon, Manju Sengar, Navin Khattry, Prashant Tembhare, Papagudi Subramanian, Sumeet Gujral

**Affiliations:** ^1^ Haematopathology Laboratory, ACTREC, Tata Memorial Centre, Navi Mumbai, India; ^2^ Adult Haematolymphoid Disease Management Group, Tata Memorial Centre, Mumbai, India; ^3^ Biostatistics, ACTREC, Tata Memorial Centre, Navi Mumbai, India; ^4^ Dept of Cytogenetics, ACTREC, Tata Memorial Centre, Navi Mumbai, India; ^5^ Homi Bhabha National Institute, Training School Complex, Mumbai, India; ^6^ Haemato-Oncology, CyteCare Cancer Hospital, Bangalore, India

**This article has been corrected:** The correct Funding information is given below:

**FUNDING**

This work was supported by the Wellcome Trust/DBT India Alliance Fellowship [grant number IA/CPHI/14/1/501485] awarded to Dr Nikhil Patkar. Initial part of the study (8 colour FCM) was funded by a Lady Tata Memorial Trust Grant awarded to Prof Navin Khattry.

Original article: Oncotarget. 2018; 9:36613-36624. https://doi.org/10.18632/oncotarget.26400

