# Supplementary material for: Clinical impact of measurable residual disease monitoring by ultradeep next generation sequencing in NPM1 mutated acute myeloid leukemia
Source: Oncotarget. 2018 Nov 27;9(93):36613–24. doi: 10.18632/oncotarget.26400 (PMC6290958; doi:10.18632/oncotarget.26400)
Supplement: Supplementary file 1 [file oncotarget-09-36613-s001.pdf]

# Clinical impact of measurable residual disease monitoring by ultradeep next generation sequencing in *NPM1* mutated acute myeloid leukemia

## SUPPLEMENTARY MATERIALS

## SUPPLEMENTARY METHODS

### Appendix

1. Clinical Outcome
2. Patient Characteristics and Cytogenetics data
3. Types of NPM1 mutations detected in our cohort
4. Comparison between NGS-VAF and RQ-PCR for Type-A *NPM1*$^{mut}$ AML MRD levels
5. *DNMT3A* mutations in NPM1mutAML
6. Comparison between NGS VAF and FCM-MRD
7. Difference in Overall Survival and Relapse Free Survival between MRD positive and negative groups
8. Correlation of mutant NPM1 variant allelic frequency detected in bone marrow and blood
9. Prognostic impact of NGS MRD using only Bone Marrow samples
10. Enrolment summary
11. Details of the PCR and sequencing primers
12. Multiparameter Flow Cytometry Analysis of MRD
    a. Panel for Flow Cytometry MRD (FCM-MRD) detection in AML
    b. Analysis of FCM-MRD Data
    c. Limit of dilution studies for FCM-MRD
13. NGS MRD validation
    a. Limit of detection testing
    b. Inter Run Coefficient of Variation at different dilutions
    c. Determination of Limit of Blank
    d. CV and Standard Deviation of the assay
    e. Coverage Metrics and QC
14. Calculation for Log Reduction between Post Induction and Post Consolidation Variant allelic frequency

*Clinical Outcome*

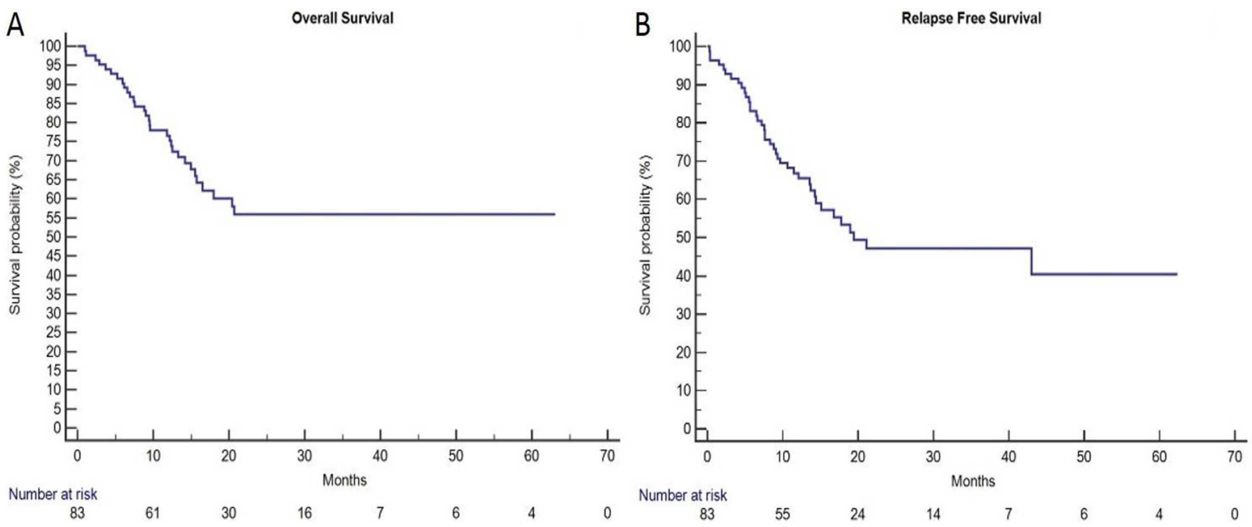


**Supplementary Figure 1:** Kaplan Meier curves for OS (**A**) and RFS (**B**) for *NPM1*$^{mut}$ AML.

## Supplementary Table 1A: Patient characteristics

| No. | Characteristics | Value |
|---|---|---|
| 1 | Sex | |
| | Male | 46 (55.4%) |
| | Female | 37 (44.6%) |
| 2 | Median age (range), years | 36.7 (18 – 62) |
| 3 | Median WBC Count (range), × $10^9$/L | 36.35 (0.1 – 270.7) |
| 4 | *FLT3*-ITD | 33 of 83 (39.8%) |
| 5 | PI NGS VAF median % (range) - 82 patients | 2.15% (0 – 36.4%) |
| 6 | PC NGS VAF % (Range) - 55 patients | 0.21% (0 – 5.8%) |

## Supplementary Table 1B: Cytogenetics data

| *NPM1_ID* | *Age* | *Sex* | *FISH*[$] | *Conventional Karyotyping*[*] |
|---|---|---|---|---|
| **NPM1-3** | **23** | **M** | Negative | **NA** |
| **NPM1-6** | **36** | **F** | Negative | **46,XX** |
| **NPM1-9** | **46** | **F** | Negative | **46,XX,del(13)(q14q21)[3]/45,X,-X [3]/46,XX[13]** |
| **NPM1-10** | **32** | **M** | Negative | **NA** |
| **NPM1-11** | **52** | **F** | Negative | **NA** |
| **NPM1-13** | **55** | **F** | Negative | **46,XX** |
| **NPM1-14** | **41** | **M** | Negative | **NA** |
| **NPM1-17** | **50** | **M** | Negative | **NA** |
| **NPM1-18** | **41** | **M** | Negative | **NA** |
| **NPM1-19** | **33** | **M** | Negative | **NA** |
| **NPM1-21** | **22** | **F** | Negative | **NA** |
| **NPM1-22** | **38** | **M** | Negative | **46,XY** |
| **NPM1-23** | **62** | **M** | Negative | **45,X,-Y[10]/45,X,-Y,t(?;9)(?;q34)[3]/44-45,XY,-Y[3],t(9)(q34)[1] [cp3]** |
| **NPM1-25** | **42** | **M** | Negative | **NA** |
| **NPM1-26** | **47** | **F** | Negative | **NA** |
| **NPM1-27** | **28** | **M** | Negative | **NA** |
| **NPM1-28** | **52** | **F** | Negative | **NA** |
| **NPM1-30** | **20** | **M** | Negative | **NA** |
| **NPM1-32** | **20** | **M** | Negative | **46,XY** |
| **NPM1-33** | **49** | **M** | Negative | **NA** |
| **NPM1-34** | **28** | **M** | **t(8;21)** | **NA** |
| **NPM1-35** | **25** | **M** | Negative | **NA** |
| **NPM1-36** | **54** | **M** | Negative | **NA** |
| **NPM1-37** | **40** | **M** | Negative | **NA** |
| **NPM1-38** | **56** | **F** | Negative | **46,XX** |
| **NPM1-39** | **54** | **M** | Negative | **NA** |
| **NPM1-40** | **37** | **M** | Negative | **47,XY, +21[10]/46,XY[3]** |
| **NPM1-41** | **25** | **M** | Negative | **NA** |
| **NPM1-42** | **50** | **M** | Negative | **46,XY** |
| **NPM1-44** | **30** | **F** | Negative | **46,XX** |
| **NPM1-45** | **44** | **F** | Negative | **NA** |
| **NPM1-46** | **44** | **M** | Negative | **46,XY** |
| **NPM1-47** | **37** | **M** | Negative | **NA** |
| **NPM1-48** | **24** | **F** | Negative | **NA** |
| **NPM1-49** | **56** | **M** | Negative | **NA** |
| **NPM1-50** | **38** | **M** | Negative | **NA** |
| **NPM1-51** | **29** | **M** | Negative | **NA** |
| **NPM1-52** | **21** | **M** | Negative | **NA** |
| **NPM1-53** | **42** | **F** | Negative | **NA** |
| **NPM1-54** | **34** | **F** | Negative | **46,XX** |
| **NPM1-55** | **21** | **F** | Negative | **NA** |
| **NPM1-56** | **36** | **F** | Negative | **NA** |
| **NPM1-57** | **19** | **M** | Negative | **46,XY, +9[2][cp2]/46,XY[17]** |
| **NPM1-58** | **43** | **M** | Negative | **NA** |
| **NPM1-59** | **52** | **F** | Negative | **NA** |
| **NPM1-60** | **57** | **M** | Negative | **NA** |
| **NPM1-61** | **41** | **F** | **NA** | **NA** |
| **NPM1-62** | **45** | **F** | Negative | **NA** |

| | | | | |
|---|---|---|---|---|
| **NPM1-63** | **48** | **F** | Negative | **NA** |
| **NPM1-64** | **32** | **M** | Negative | **NA** |
| **NPM1-65** | **45** | **M** | Negative | **46,XY** |
| **NPM1-67** | **44** | **F** | Negative | **NA** |
| **NPM1-68** | **37** | **F** | **t(9;22)** | **NA** |
| **NPM1-69** | **21** | **M** | Negative | **46,XY** |
| **NPM1-70** | **32** | **F** | Negative | **NA** |
| **NPM1-71** | **24** | **M** | Negative | **NA** |
| **NPM1-74** | **45** | **F** | Negative | **46,XX, ?inv(10) (p13;q22)[10] / 46,XX [7]** |
| **NPM1-75** | **25** | **F** | Negative | **46,XX** |
| **NPM1-77** | **26** | **F** | Negative | **46,XX** |
| **NPM1-78** | **37** | **F** | Negative | **46-47,XX, i(Xq)[2],del(9q11)[2],del(11q23)[2],+21[3][cp7]/** |
| **NPM1-79** | **41** | **F** | Negative | **NA** |
| **NPM1-80** | **18** | **M** | Negative | **44-45,XY, random abnormalities[2]/ 46,XY[13]** |
| **NPM1-81** | **58** | **M** | Negative | **NA** |
| **NPM1-82** | **32** | **M** | Negative | **NA** |
| **NPM1-83** | **22** | **M** | Negative | **46,XY, del(11)(q13)[2]/ 43-46,XY,del(5q)[2], -8[3], +8[2], t(13;?)(p11;?)[2],+21[2][cp5]/ 46,XY[6]** |
| **NPM1-84** | **20** | **F** | Negative | **NA** |
| **NPM1-85** | **30** | **F** | Negative | **46,XX,del(5)(q13.1q13.1)[4]/ 46,XX,t(Xp)[2][cp2]/** |
| **NPM1-86** | **32** | **M** | **Trisomy 8** | **44-46, XY, del (5) (q? 15/q22)[1], random aberrations[4]/ 46, XY[11]** |
| **NPM1-87** | **22** | **F** | Negative | **44-46,XX,/X, -X[3], -21[3],+mar1[2][cp5]/ 46, XX[10]** |
| **NPM1-89** | **45** | **F** | Negative | **46,XX,t(11;?)(q23;?)[5]/45,XX,t(11;?)[3][cp3]/46,XY[11]** |
| **NPM1-90** | **19** | **M** | **t(8;21)** | **45,XY,t(8;21)(q22;q22),ins(13)(?;q12.2)[3]/ 45,XY,-Y[3], t(8;21)[13],+8[5],ins(13)[11],-13[2],-14[8],-17[3],+21[2][cp14]/ 46,XX,[1]** |
| **NPM1-91** | **24** | **M** | **trisomy 3** | **NA** |
| **NPM1-92** | **23** | **F** | **inv(16),del(7q)** | **NA** |
| **NPM1-93** | **49** | **F** | Negative | **NA** |
| **NPM1-94** | **30** | **M** | Negative | **46,XY** |
| **NPM1-96** | **41** | **M** | Negative | **47-49,XY, +4[2],-9[2],+12[2], +22[6][cp6]/ 46,XY[12]** |
| **NPM1-97** | **41** | **F** | Negative | **NA** |
| **NPM1-98** | **54** | **M** | **NA** | **NA** |
| **NPM1-99** | **38** | **F** | Negative | **NA** |
| **NPM1-101** | **25** | **F** | **del (5q)** | **46,XX,i(3q)[3]/46,XX, del(5)(q33)[3]/46,XX, i(3q),del(5)(q33)[2]/45-46,XX, i(3q)[3],del(5q)[1],-18[3][cp4]46,XX[8]** |
| **NPM1-103** | **24** | **M** | Negative | **46,XY** |
| **NPM1-104** | **49** | **M** | **Trisomy 8** | **NA** |
| **NPM1-111** | **43** | **F** | Negative | **NA** |

(NA- not available due to metaphase failure, *- As per International System for Cytogenetic Nomenclature, $- FISH was done for t(8;21), inv(16)/t(16;16), t(v;11q23): MLL , deletion (5q), deletion (7q), Trisomy 8, TP53 deletion, t(9;22).

## Supplementary Table 2: Types and Frequency of *NPM1* mutations detected in our cohort

| Chromosome | Start | Insertion | *NPM1* Allele Type | Frequency (%) |
|---|---|---|---|---|
| chr5 | 170837543 | TCTG | A | 69.14 |
| chr5 | 170837545 | TGCA | B | 7.41 |
| chr5 | 170837545 | TGCG | C | 1.23 |
| chr5 | 170837544 | CTGC | D | 7.41 |
| chr5 | 170837546 | GCAG | G | 2.47 |
| chr5 | 170837545 | TGCT | I | 1.23 |
| chr5 | 170837546 | GCCG | J | 1.23 |
| chr5 | 170837546 | GCCA | K | 1.23 |
| chr5 | 170837545 | TGTT | L | 1.23 |
| chr5 | 170837545 | TGTA | R | 3.7 |
| chr5 | 170837546 | GTAG | Z | 2.47 |
| chr5 | 170837557 | GCCA | Novel | 1.23 |

***Comparison between NGS-VAF and RQ-PCR for Type-A NPM1*** $^{mut}$ **AML *MRD levels***

at

C-

of

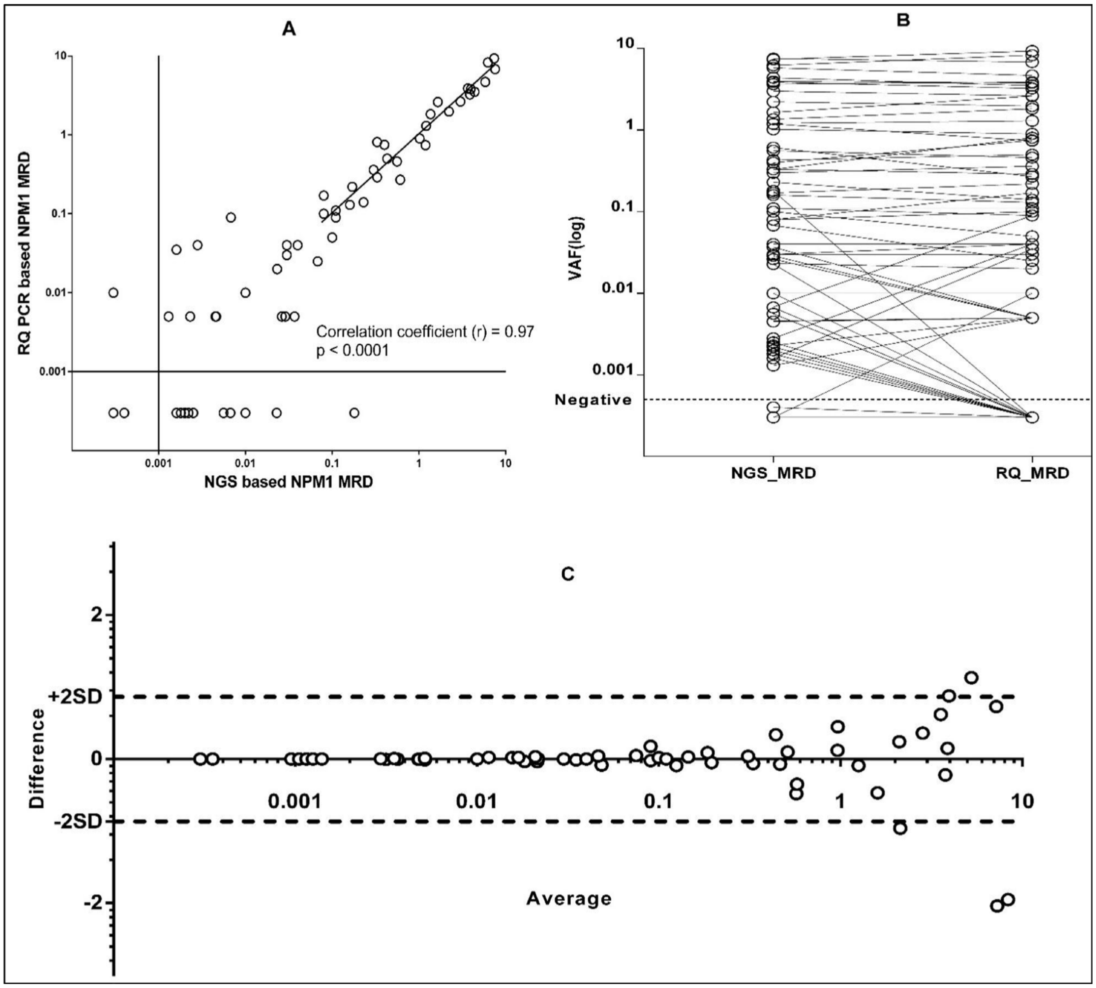


**Supplementary Figure 2:** (**A**) Correlation between real time PCR and NGS assays at MRD time points, (**B**) Comparison between NGS VAF and RQ-PCR MRD in *NPM1*$^{mut}$AML, (**C**) Difference vs. average: Bland-Altman analysis of *NPM1* NGS MRD as compared to RQ PCR. (Replacement approach was used for values below limit of detection for log calculation).

## Supplementary Table 3: Types and Dynamics of *DNMT3A* mutations with respect to *NPM1*$^{mut}$AML at different treatment time points

| Sample ID | HGVS genomic change | HGVS protein change | HGVS Coding DNA Change | Exon | DNMT3A VAF at Baseline | Corresponding NPM1 VAF at Baseline | DNMT3A VAF at PI | Corresponding NPM1 VAF at PI | DNMT3A VAF at PC | Corresponding NPM1 VAF at PC |
|---|---|---|---|---|---|---|---|---|---|---|
| NPM1-3 | 2:g.25463266G>A | ENSP00000321117p.Pro743Ser | ENST00000321117.5c.2227C>T | exon19 | 42.34% | 49.26% | Negative | Negative | NA | NA |
| NPM1-6 | 2:g.25457243G>A | ENSP00000321117p.Arg882Cys | ENST00000321117.5c.2644C>T | exon23 | 47.89% | 42.55% | 13.18% | 1.3577% | NA | NA |
| NPM1-13 | 2:g.25463266G>A | ENSP00000321117p.Pro743Ser | ENST00000321117.5c.2227C>T | exon19 | 42.41% | 38.94% | 22.23% | 0.5586% | NA | NA |
| NPM1-18 | 2:g.25457242C>T | ENSP00000321117p.Arg882His | ENST00000321117.5c.2644C>T | exon23 | 46.18% | 41.45% | Negative | 1.2116% | Negative | Negative |
| NPM1-32 | 2:g.25469170T>G | ENSP00000321117p.Asn430His | ENST00000321117.5c.1288A>C | exon11 | 4.82% | 46.14% | Negative | 0.6685% | NA | NA |
| NPM1-45 | 2:g.25457243G>A | ENSP00000321117p.Arg882Cys | ENST00000321117.5c.2644C>T | exon23 | 50.99% | 24.84% | Negative | 0.1693% | Negative | Negative |
| NPM1-46 | 2:g.25464460C>A | ENSP00000321117p.Gly685Trp | ENST00000321117.5c.2053G>T | exon17 | 1.87% | 36.91% | 4.35% | 0.232% | 5.08% | 0.0037% |
| NPM1-47 | 2:g.25457242C>T | ENSP00000321117p.Arg882His | ENST00000321117.5c.2644C>T | exon23 | 47.11% | 31.53% | Negative | 0.0031% | Negative | 0.1863% |
| NPM1-50 | 2:g.25469584_ 25469584insT | ENSP00000321117p.Thr395fs | ENST00000321117.5c.1184dupA | exon10 | 34.48% | 35.83% | Negative | 0.0798% | Negative | 0.0152% |
| NPM1-54 | 2:g.25470577delT | ENSP00000321117p.Lys299fs | ENST00000321117.5c.897delA | exon8 | 47.50% | 17.85% | Negative | 0.0771% | NA | NA |
| NPM1-62 | 2:g.25463308G>A | ENSP00000321117p.Arg729Trp | ENST00000321117.5c.2185C>T | exon19 | 23.49% | 23.52% | 25.36% | 0.0791% | NA | NA |
| NPM1-78 | 2:g.25457243G>A | ENSP00000321117p.Arg882Cys | ENST00000321117.5c.2644C>T | exon23 | 43.48% | 28.94% | 38.40% | 0.0326% | 46.49% | 0.0025% |
| NPM1-82 | 2:g.25457242C>T | ENSP00000321117p.Arg882His | ENST00000321117.5c.2644C>T | exon23 | 43.40% | 39.97% | Negative | 0.1728% | Negative | Negative |
| NPM1-84 | 2:g.25469170T>G | ENSP00000321117p.Asn430His | ENST00000321117.5c.1288A>C | exon11 | 3.35% | 25.85% | Negative | 0.0724% | Negative | Negative |
| NPM1-97 | 2:g.25457242C>T | ENSP00000321117p.Arg882His | ENST00000321117.5c.2644C>T | exon23 | 47.70% | 42.35% | 6.39% | 4.2246% | 6.15% | 3.9617% |
| NPM1-98 | 2:g.25457149_ 25457149insAC | ENSP00000321117p. X913delinsC | ENST00000321117.5c.2736_2 737dupGT | exon23 | 8.84% | 13.51% | 3.81% | 0.3266% | NA | NA |

(HGVS- Human Genome Variation Society, PI- Post Induction, PC- Post First consolidation, VAF- Variant Allelic Frequency, NA- Not available).

*Comparison between NGS VAF and FCM-MRD*

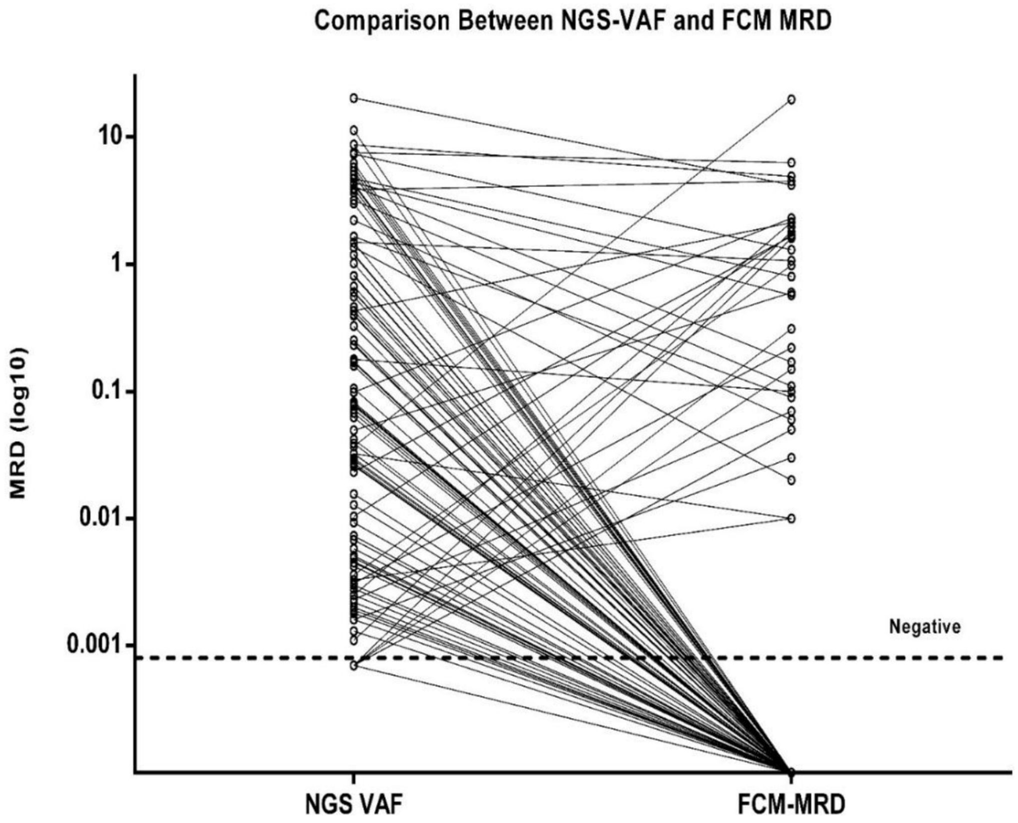


**Supplementary Figure 3: Comparison of NGS variant allele frequencies with corresponding FCM-MRD value.** No significant correlation was detected between the two methods.

*Difference in Overall Survival and Relapse Free Survival between MRD positive and negative groups*

## Supplementary Table 4: Survival statistics of patients stratified as per MRD results

| | Overall Survival (OS) | | Relapse Free Survival (RFS) | |
|---|---|---|---|---|
| **PI FCM-MRD** | | | | |
| MRD Negative | Mean OS: 43.9 months; 95% CI (37.0–50.9 months), Median OS: not reached | ***p* = 0.006** | Mean RFS: 35.7 months; 95% CI (28.1–43.4 months), Median RFS: 43.0 months; 95% CI (17.7–43.03 months), | ***p* = 0.0097** |
| MRD Positive | Mean OS: 26.8 months; 95% CI (16.4–37.2 months) Median OS: 14.9 months; 95% CI (9.0 to 20.4 months) | | Mean RFS: 23.1 months; 95% CI (13.2–33.0 months); Median RFS: 9.7 months; 95% CI (7.2 to 15.1 months) | |
| **NGS-MRD** | | | | |
| MRD Negative | Mean OS: 42.2 months; 95% CI (33.1–51.2 months), Median OS: not reached | ***p* = 0.0009** | Mean RFS: 38.5 months; 95% CI (29.4–47.5 months), Median RFS: 43.0 months; 95% CI (16.8–43.0 months), | ***p* < 0.0001** |
| MRD Positive | Mean OS: 19.4 months; 95% CI (8.1–30.8 months) Median OS: 12.1 months; 95% CI (8.8 to 20.4 months) | | Mean RFS: 11.3 months; 95% CI (3.9–18.7 months); Median RFS: 7.2 months; 95% CI (5.1 to 9.3 months) | |

CI, confidence interval.

### *Correlation of mutant NPM1 variant allelic frequency detected in bone marrow and blood*

There were 19 patients in which bone marrow (BM) and peripheral blood (PB) samples were available at same time point. There was a reasonable correlation between mutant *NPM1* VAF in BM and PB. (Spearman's $r$ = 0.897, $P < 0.0001$). (Supplementary Figure 4A) Based on these results we also calculated that there was a mean 0.7 Log (median 0.41Log) difference between *NPM1* MRD measurements in the BM and blood. (Supplementary Figure 4B).

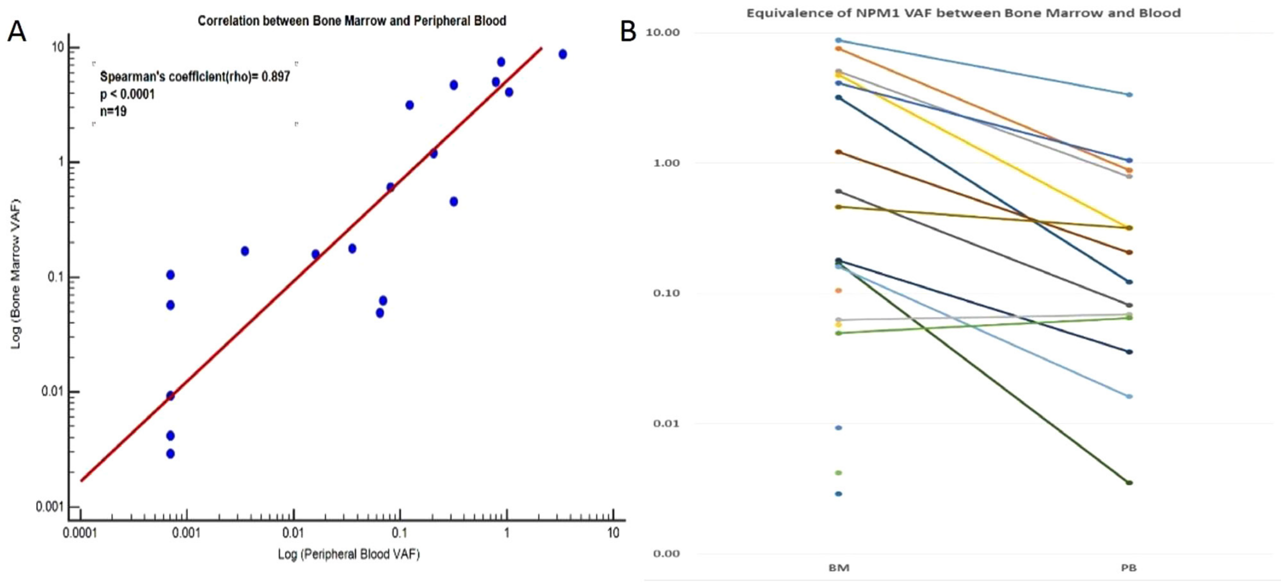


**Supplementary Figure 4:** Matched samples of BM and PB (**A**) The plot showing correlation between the peripheral blood and bone marrow using Pearson's index. (**B**) Mean difference of 0.7 log was observed between matched samples.

### *Prognostic impact of NGS MRD using only Bone Marrow samples*

We excluded paired cases in which NGS was done on peripheral blood samples at post consolidation time point as bone marrow sample was not available to determine if there was a bias in the results due to a combination of BM and PB. As can be seen in Supplementary Figure 5 NGS MRD was still significantly predictive of outcome.

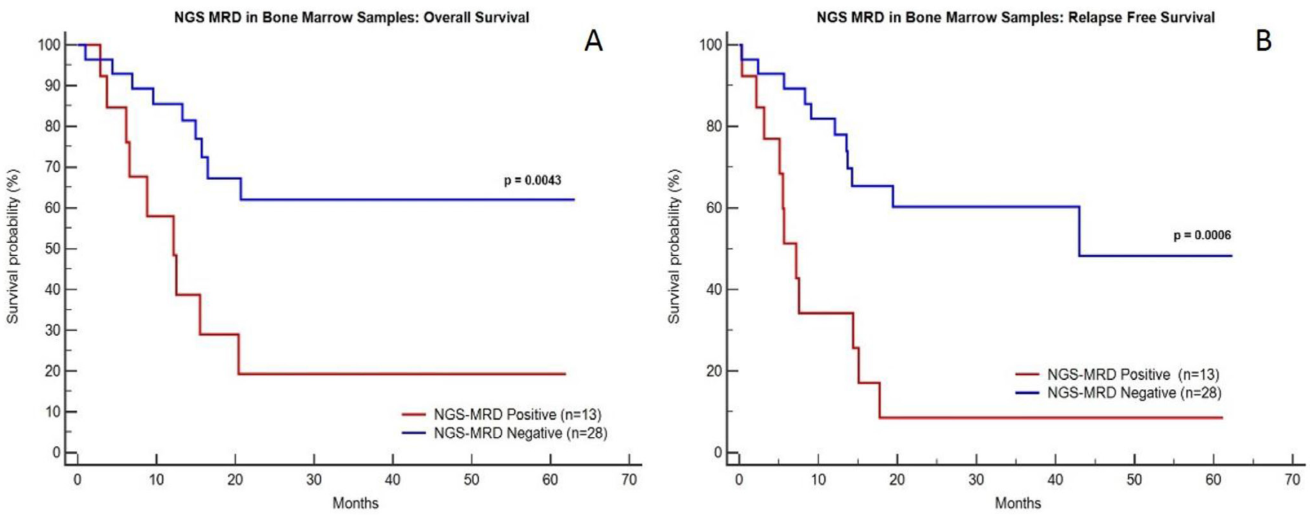


**Supplementary Figure 5: NGS MRD in only bone marrow samples.** MRD positivity is shown to be significantly predictive of inferior OS (**A**) and RFS (**B**) even when only BM samples were considered.

*Enrolment summary*

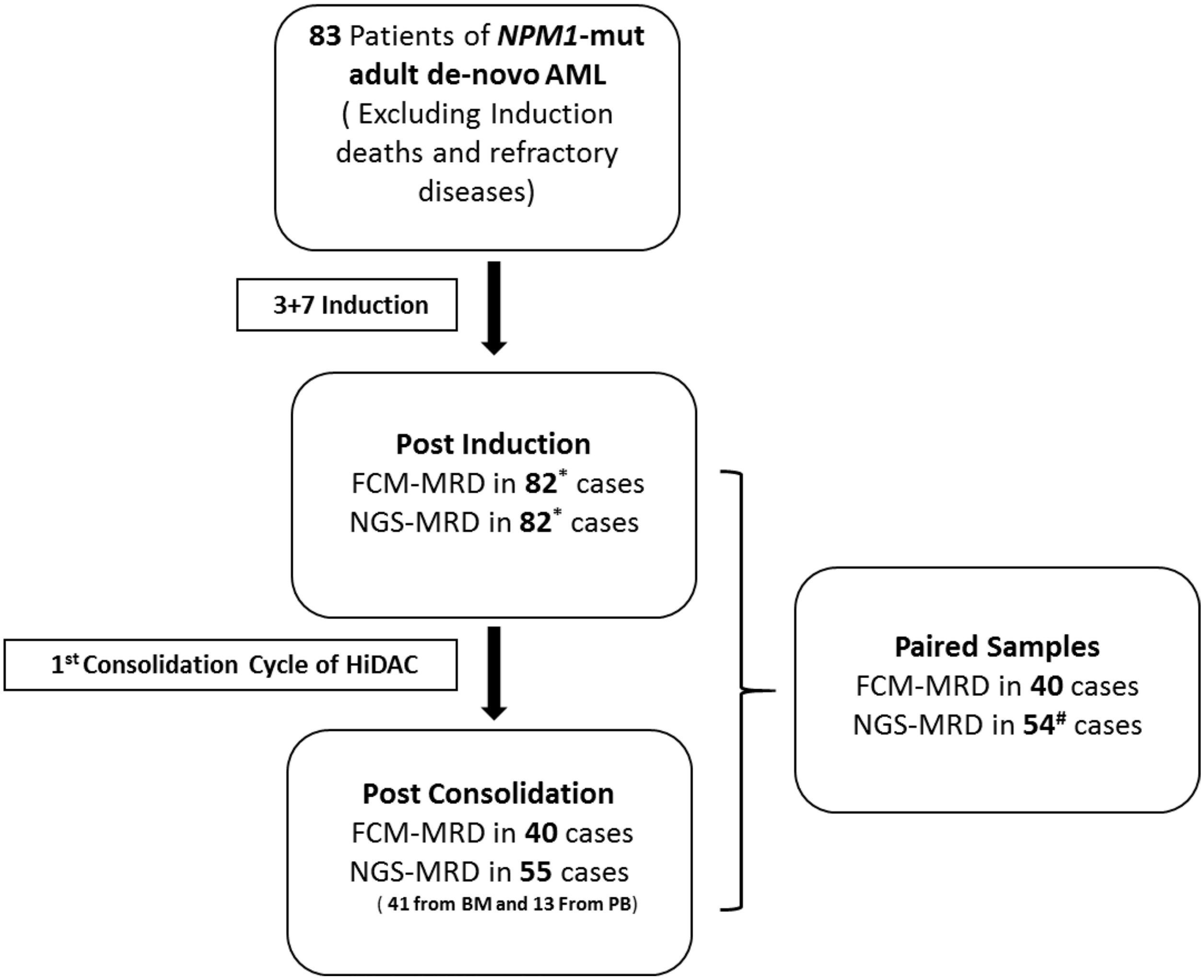


**Supplementary Figure 6: Characteristics of the patient cohort.** (*Out of 83 cases at Post Induction, in one case FCM data was not available and, in another case, DNA was not available for NGS MRD. #Of 55 cases sequenced at Post Consolidation, in one case Post Induction Sample was not available for NGS MRD, hence there were 54 Paired NGS -MRD cases).

*Details of primers used for next generation sequencing*

**Supplementary Table 5: Adapter tagged locus specific and sequencing primers**

| *NPM1* Primers | Sequences |
|---|---|
| **Forward Primer*** | 5′AATGATACGGCGACCACCGAGATCTACAC**XXXXXXXXXX**TATGGTGCCTGTAAACACGGTAGGGAAAGTTCTC-3′ |
| **Reverse primer*** | 5′CAAGCAGAAGACGGCATACGAGAT**XXXXXXXXXX**AGTCAGTCAGTCTGTCTATGAAGTGTTGTGGTTCC-3′ |
| **Read 1 Primer** | 5′-TATGGTGCCTGTAAACACGGTAGGGAAAGTTCTC-3′ |
| **Read 2 primer** | 5′AGTCAGTCAGTCTGTCTATGAAGTGTTGTGGTTCC-3′ |
| **Index Primer** | 5′-GGAACCACAACACTTCATAGACAGACTGACTGACT-3′ |

*XXXXXXXXXX represents 10 bp sample specific index sequence.

## DETECTION OF MRD USING MULTICOLOUR FLOW CYTOMETRY

*Panel for Flow Cytometry MRD (FCM-MRD) detection in AML*

**Supplementary Table 6: Panel of antibodies and their clones used in three tubes 8 colour MRD assay**

| | FITC | PE | PerCP Cy5.5 | PE Cy7 | APC | APC H7 | BV450/ BV421 | BV510 |
|---|---|---|---|---|---|---|---|---|
| TUBE1 | CD38 | CD123 | CD33 | CD117 | CD34 | CD45 | CD36 | HLA-DR |
| Clone | T16 | 9F5 | P67.6 | 104D2D1 | 581 | J.33 | CB38 | L243 |
| TUBE2 | CD56 | CD13 | CD33 | CD19 | CD34 | CD45 | CD7 | HLA-DR |
| Clone | NCAM16.2 | SJ1D1 | P67.6 | SJ25C1 | 581 | J.33 | M-T701 | L243 |
| TUBE3 | CD16 | CD11b | CD15 | CD13 | CD34 | HLA-DR | CD117 (BV421) | CD45 |
| Clone | DJ130C | ICRF44 | HI98 | Immu103.44 | 581 | G46-6 | YB5.B8 | HI30 |

**Supplementary Table 7: Panel of antibodies and their clones used in two tubes 10 colour MRD assay**

| | FITC | PE | ECD | PerCP Cy 5.5 /PC5.5 | PECy7/ PC7 | APC | APC AF700 | APC AF750 | BV421 | BV510 |
|---|---|---|---|---|---|---|---|---|---|---|
| TUBE1 | CD14 | CD123 | CD64 | CD33 | CD117 | CD34 | CD45 | CD38 | CD36 | HLA-DR |
| Clone | RM052 | 9F5 | CLONE 22 | P67.6 | 104D2D1 | 581 | J.33 | LS198.4.3 | CB38 | L243 |
| TUBE2 | CD15 | CD13 | CD19 | CD34 | CD56 | CD7 | CD45 | CD11b | CD117 | HLA-DR |
| Clone | 80H5 | SJ1D1 | J3-119 | 581 | N901 (HLDA6) | 8H8.1 | J.33 | BEAR 1 | YB5.B8 | L243 |

***Analysis of FCM-MRD Data***

Familiarity with normal myeloid maturation and standardization of normal templates was achieved on stressed regenerative bone marrows (for e.g. ALL post induction that were MRD negative) using the antibody panels as seen above. A representative case of *NPM1*-mut AML which is positive at the post induction time point is shown in Supplementary Figure 7.

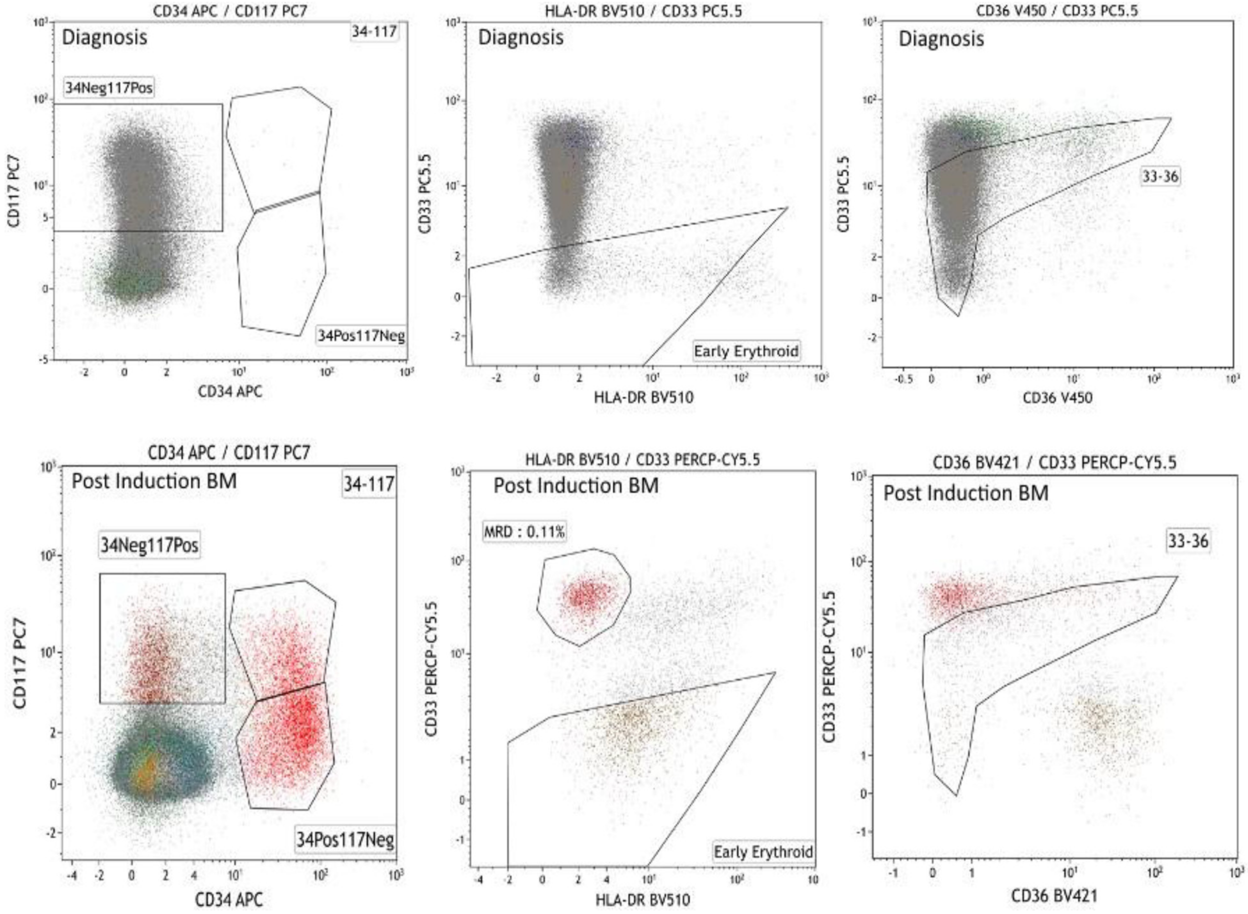


**Supplementary Figure 7: An example of FCM-MRD detection in a typical *NPM1*-mut AML.** The panel on top shows that the blasts (grey) are CD34 negative, heterogeneous for CD117 and negative for HLA-DR and CD36. The panel below shows that these blasts are present (brown) at a frequency of 0.11% (of all nucleated cells) in the post induction BM. Normal myeloid progenitors are coloured red.

*Limit of dilution studies for FCM-MRD*

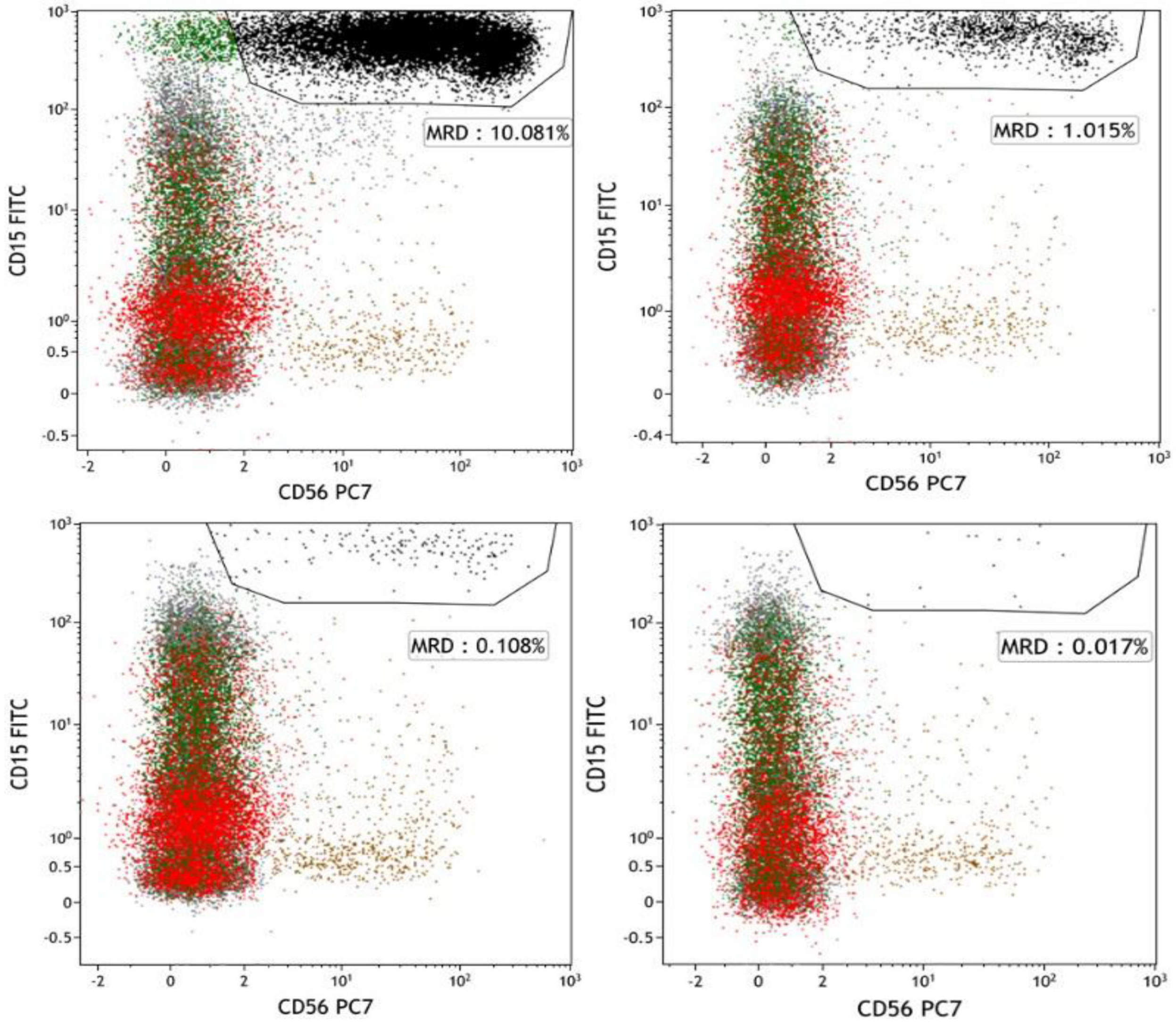


**Supplementary Figure 8: Limit of detection testing for the AML-MRD assay.** An OCI-AML3 cell line was serially diluted in a normal bone marrow. The analysis shows that these cells can be detected based on an abnormal immunophenotype (CD56 and CD16 positive, in black) at a frequency of 0.01%.

## NGS MRD ASSAY VALIDATION USING ULTRA-DEEP SEQUENCING

### *Limit of detection testing*

For establishment of limit of detection, we serially diluted an OCI-AML3 cell line (known to harbour a type-A *NPM1* mutation) in normal bone marrow ranging from 10% to 0.001%. As the Supplementary Figure 9 demonstrates we could successfully detect *NPM1* mutation at 1:100,000 dilution using NGS. Thus, for NGS 0.001% was the limit of detection of the assay (LOD) as already established. [2] Based on the results of these two experiments, we obtained inter run CV as can be seen in Supplementary Table 8.

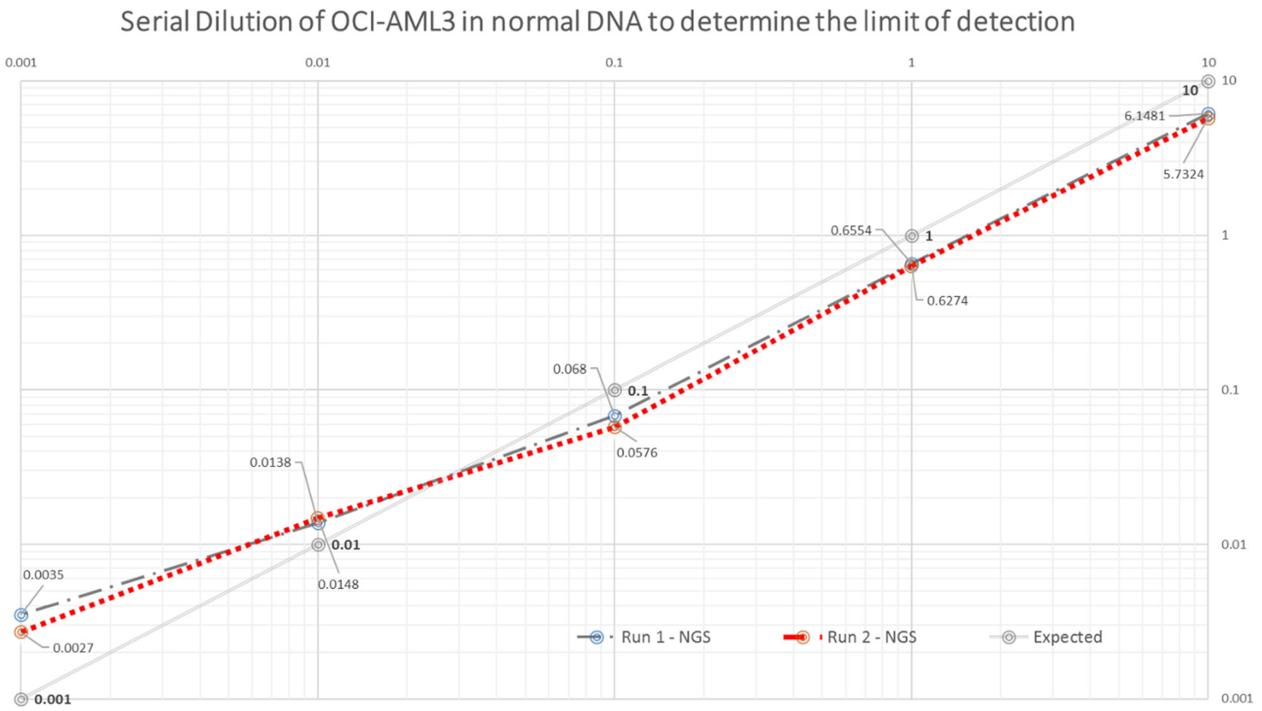


**Supplementary Figure 9: Results of serially diluted OCI-AML3 in normal BM sample.** Observed variant allele frequencies (in percentages) of two dilution experiments are plotted against expected frequencies.

***Inter Run Coefficient of Variation at different dilutions***

**Supplementary Table 8: Inter run coefficient of variation at different dilutions**

| Percentage of *NPM1* mutant cells | Inter Run CV |
|---|---|
| 10% | 0.05 |
| 1% | 0.03 |
| 0.1% | 0.12 |
| 0.01% | 0.05 |
| 0.001% | 0.18 |

***Determination of Limit of Blank***

To determine the specificity of the assay we performed a limit of blank (LOB) study. For LOB, DNA was extracted from 30 normal peripheral blood samples and were subjected to ultradeep NGS to check that false calls of *NPM1* mutations were not made. LOB analysis did not show any four (or more) bp insertions. The average sequencing coverage for this experiment was 410,342X (average for 30 samples). This proves the specificity of the assay for determination of MRD in *NPM1*-mut AML.

*CV and Standard Deviation of the assay*

With each run, two *NPM1* mutation positive precision controls (OCI-AML3 in NA12878 DNA) were assayed (0.2% and 0.02%). Data were plotted in control chart for high precision (0.2%) and low precision (0.02%) controls as seen in Supplementary Figure 10. The CV for these low precision controls were 15.36% and 15.49% respectively.

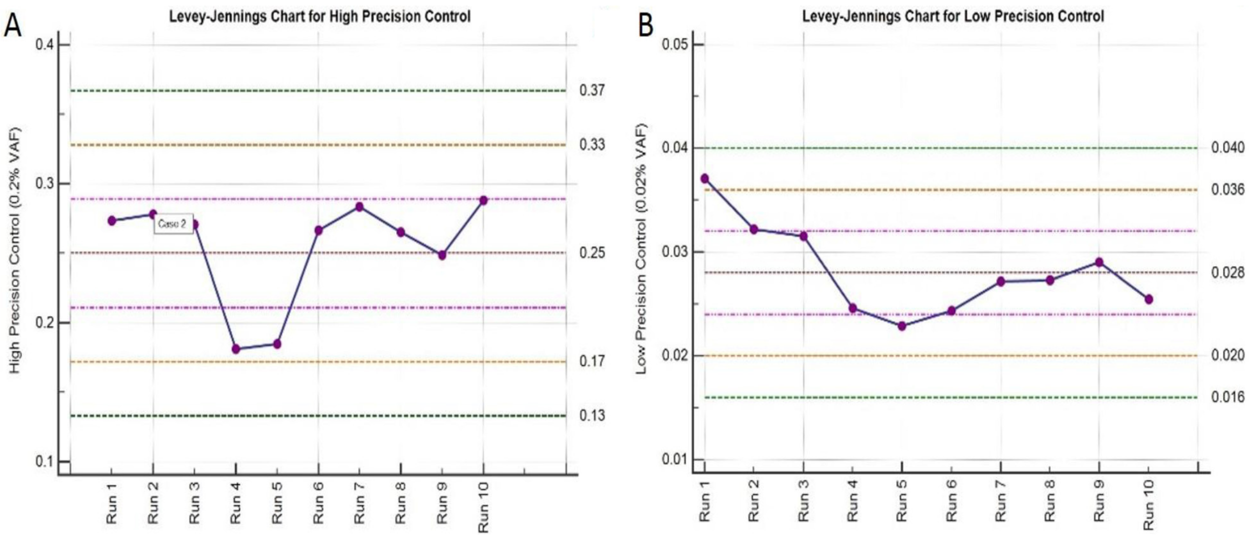


**Supplementary Figure 10: Levey-Jennings plots of precision controls.** (**A**) High Precision Control (0.2% VAF) and (**B**) Low Precision Control (0.02% VAF).

*Coverage Metrics and QC*

The average coverage for MRD samples (PI and PC, $n$ = 137) was 565,808x and median coverage was 551,227x. It was made sure that negative calls were made only if the sequencing coverage was more than 2,00,000x. We also added an NA12878 control (Coriell Bio Repository, Camden, NJ, USA) with every run to ensure that false positives variant calls were not made.

*Calculation for Log Reduction between Post Induction and Post Consolidation Variant allelic frequency*

While calculating log values of *NPM1* mutant allelic frequencies, replacement approach was used for values below limit of detection (i.e. less than 0.001%). As per Croghan *et.al*. [3] LOD/√2 is the best choice of replacement for left censored data. Hence in our cohort while calculating log difference between 'post induction' and 'post consolidation' time points, the cases in which original VAF values were less than 0.001% were substituted by a constant value of LOD/√2 i.e. (0.0007).

## REFERENCES

1. Döhner H, Estey E, Grimwade D, Amadori S, Appelbaum FR, Büchner T, Dombret H, Ebert BL, Fenaux P, Larson RA, Levine RL, Lo-Coco F, Naoe T, et al. Diagnosis and management of AML in adults: 2017 ELN recommendations from an international expert panel. Blood. 2017; 129:424–47. https://doi.org/10.1182/blood-2016-08-733196.
2. Salipante SJ, Fromm JR, Shendure J, Wood BL, Wu D. Detection of minimal residual disease in NPM1-mutated acute myeloid leukemia by next-generation sequencing. Mod Pathol. Nature Publishing Group; 2014; 27:1438–46. https://doi.org/10.1038/modpathol.2014.57.
3. Croghan CW, Egeghy PP. Methods of Dealing With Values Below the Limit of Detection Using Sas. South SAS User Gr. 2003; 5.
